# Typing African Relapsing Fever Spirochetes

**DOI:** 10.3201/eid1111.050483

**Published:** 2005-11

**Authors:** Julie Christine Scott, David Julian Maurice Wright, Sally Jane Cutler

**Affiliations:** * Imperial College of Science, Technology and Medicine, South Kensington, London, United Kingdom; † Veterinary Laboratories Agency, Weybridge, Surrey, United Kingdom

**Keywords:** Tickborne relapsing fever, Louseborne relapsing fever, intragenic spacer molecular typing, spirochetes, *Borrelia*, research

## Abstract

Relapsing fever *Borrelia* spp. challenge microbiologic typing because they possess segmented genomes that maintain essential genes on large linear plasmids. Antigenic variation further complicates typing. Intergenic spacer (IGS, between 16S–23S genes) heterogeneity provides resolution among Lyme disease–associated and some relapsing fever spirochetes. We used an IGS fragment for typing East African relapsing fever *Borrelia* spp. *Borrelia recurrentis* and their louse vectors showed 2 sequence types, while 4 *B. duttonii* and their tick vectors had 4 types. IGS typing was unable to discriminate between the tick- and louseborne forms of disease. *B. crocidurae*, also present in Africa, was clearly resolved from the *B*. *recurrentis*/*B*. *duttonii* complex. IGS analysis of ticks showed relapsing fever *Borrelia* spp. and a unique clade, distant from those associated with relapsing fever, possibly equivalent to a novel species in ticks from this region. Clinical significance of this spirochete is undetermined.

## Abstract 2

Sequencing distinguished relapsing fever from other borrelial species but not *B*. *duttonii* from *B*. *recurrentis.*

Both tickborne and louseborne forms of relapsing fever, caused by borrelial spirochetes, are substantial causes of illness and death in regions of East Africa. In Tanzania and Ethiopia, for example, these infections are endemic, with much of the population living in close proximity with the disease vectors. Indeed, surveys of traditional Tanzanian "Tembe" dwellings have shown that up to 88% are infested with ticks of the *Ornithodoros moubata* complex (*1*). These ticks are vectors for *Borrelia duttonii*, the cause of tickborne relapsing fever in East Africa. In disease-endemic regions, tickborne relapsing fever is one of the top 10 diseases associated with deaths in those <5 years of age. The louseborne form of the disease is caused by *B. recurrentis* and is transmitted by the human clothing louse, *Pediculus humanus*. In regions where louseborne relapsing fever is endemic, such as Ethiopia, this disease contributes substantially to human disease prevalence, particularly during the rainy season. Incidence data are largely lacking; the diagnosis typically relies on demonstration of spirochetes in blood films of samples taken from febrile patients. Clinical signs and symptoms overlap with those of other prevalent diseases such as malaria, which may lead to inaccurate diagnoses.

Other *Borrelia* spp. have been associated with relapsing fever in Africa; for example, *B*. *crocidurae* is associated with this disease in West Africa (*2*). However, this spirochete has an established reservoir in rodent populations that also serve as hosts for the *Ornithodoros erraticus* ticks. Recent reports have also established additional spirochetes in tick vectors (*3*) and in human blood samples (*4*); however, their clinical relevance has yet to be defined.

To determine the population structure within these spirochetes and to assess the role of newly described spirochetes, appropriate microbiologic tools must be used. Many widely used techniques for molecular typing have been applied to *Borrelia* with variable success (*5**,**6*). Of concern when these methods are used for characterizing these spirochetes is the organisms' ability to undergo multiphasic antigenic variation, which can be associated with large inter- or intraplasmidic recombinations/duplications (*7**,**8*). Indeed, this ability is a likely cause of the variability in genomic organization of these spirochetes, which possess segmented genomes (*9**–**11*) composed of giant linear and circular plasmids.

Although conventional approaches, such as sequencing of 16SRNA gene, have provided useful information on population structure among the *Borrelia* spp., this gene only provides low discriminatory resolution among African relapsing fever spirochetes (*2**,**12*). Others have used the flagellin gene as a target for population structure (*5**,**13*); however, as with 16SRNA, these methods, although useful for interspecies comparisons, are of limited value for discrimination of African relapsing fever spirochetes and for intraspecies analysis. A partial intergenic spacer region (IGS; *rrs* [16S rRNA]-*ileT* [tRNA]) has recently been used for typing Lyme-associated *Borrelia* spp., together with other genetic loci (*14*). Additionally, IGS fragment typing proved valuable for differentiating relapsing fever spirochetes (*15*). However, these researchers only applied this typing to US strains and to nonpathogenic relapsing fever spirochetes from Sweden. We applied this method to isolates, blood samples, ticks, and lice collected in Tanzania and Ethiopia.

## Materials and Methods

### Blood Samples

Blood samples from Tanzania were collected into glass capillary tubes after fingerprick of spirochetemic patients attending Mvumi Hospital, Dodoma, Tanzania. In Ethiopia, samples were collected from persons who were hospitalized with louseborne relapsing fever at the Black Lion Hospital in Addis Ababa. Total DNA was extracted from these samples by using a QIAamp DNA blood mini kit (Qiagen, Crawley, UK).

### Isolates

Borrelial isolates were obtained from blood samples drawn from patients with clinical cases of relapsing fever as previously described (*16**–**18*). *Borrelia* spp. were grown in Barbour-Stoenner-Kelly medium (BSKII) (*19*) incubated at 33°C to stationary phase. Spirochetes were collected by centrifugation, washed with 0.1 mmol. phosphate-buffered saline, and DNA was prepared by using phenolchloroform extraction. A total of 6 cultivated *B*. *duttonii* isolates (Ly, La, Lw, Ma, Ku, and Wi) and 18 *B*. *recurrentis* isolates (A1–A18) were investigated. Cultures were diluted in BSKII medium, and the one with the lowest growth was added to media from which DNA was extracted. Because these spirochetes are fastidious, recovery from a single cell cannot be guaranteed. For comparison, other *Borrelia* spp. analyzed included Nearctic relapsing fever strains *B*. *hermsii* (HS1), *B*. *turicatae*, and *B*. *parkeri*; West African *B*. *crocidurae*; and Lyme borreliosis strain *B*. *burgdorferi* sensu stricto (B31).

### Lice

Lice were collected from the clothing of patients with louseborne relapsing fever, kept in 70% ethanol, and transported to the United Kingdom (import license not required). Total DNA was extracted by using a DNeasy Tissue kit (Qiagen) from pools of 4 to 6 lice.

### Ticks

Ticks were collected from traditional dwellings in 4 villages, Mvumi Makulu (MK), Iringa Mvumi (IM), Ikombolinga (IK), and Mkang'wa (MA), in the Dodoma Rural District, central Tanzania. Scoops of earth were collected and passed through a sieve, and ticks were collected into containers containing 70% ethanol. Dead ticks were imported under license (AHZ/2074A/2001/13) and heat inactivated (>80°C for 30 min) before DNA extraction. After manual homogenization of 1 to 6 ticks with a sterile pestle, samples were digested overnight in SNET lysis buffer (20 mmol/L Tris-HCl pH 8.0, 5 mmol/L EDTA, 400 mmol/L NaCl, 1% sodium dodecyl sulfate, 55°C), supplemented with proteinase K (400 μg/mL final concentration). Debris was pelleted, lysate was transferred to a fresh tube, and total DNA extracted by using standard phenolchloroform extraction or the automated MagnaPure DNA extraction robot with the LC DNA isolation kit II for tissues (Roche, Lewes, UK). DNA extracted from 2 purification rounds was pooled, concentrated, and resuspended in sterile distilled water.

### PCR

A *Borrelia*-specific nested polymerase chain reaction (PCR) designed to amplify the 16S–23S IGS region was used (14). The outer primers were anchored in the 3´ end of the *rrs* gene and the *ileT* genes, respectively (5´-GTATGTTTAGTGAGGGGGGTG-3´ and 5´-GGATCATAGCTCAGGTGGTTAG-3´ for forward and reverse, respectively, while the inner nested primers were 5´-AGGGGGGTGAAGTCGTAACAAG-3´ and 5´-GTCTGATAAACCTGAGGTCGGA-3´, again for forward and reverse). Amplicons were resolved with 1% agarose gels, bands were excised, and DNA was purified by using a Wizard SV gel and PCR clean-up system (Promega, Southhampton, UK).

### DNA Sequencing

Purified DNA was sequenced directly or cloned into pGEMT-easy (Promega) and sequenced. Sequencing reactions were performed according to manufacturer's recommendations by using BigDye terminator v3.1 cycle sequencing kit (Applied Biosystems, Warrington, UK) and analyzed on an Applied Biosystems Genetic Analyzer (Applied Biosystems, Foster City, CA, USA).

### Data Analysis

Nucleotide sequences were analyzed by using Chromas (version 1.45) and DNA Star software (Lasergene 6). Multiple alignments were performed by using ClustalW. Results produced by IGS fragment typing were compared with those obtained using the *rrs* gene with sequences held in GenBank.

### Phylogenetic Trees

The phylogenetic relationships of sequence data were compared by using Mega software (version 3) and neighbor-joining methods for compilation of the tree. A bootstrap value of 250 was used to determine confidence in tree-drawing parameters.

## Results

### Clinical Isolates

**Figure 1:**
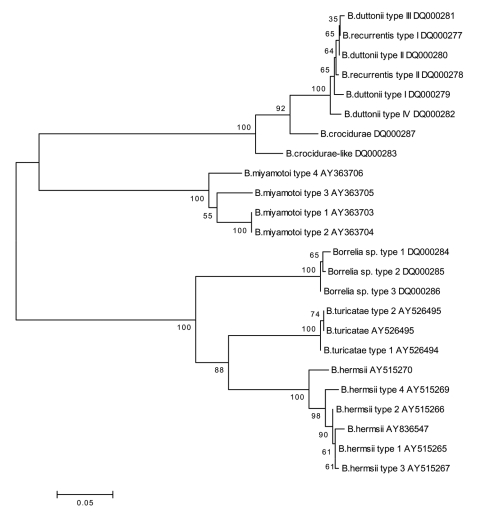
Neighbor-joining phylogenetic tree (bootstrap value 250) showing clustering of intergenic spacer (IGS) fragment generated within this study and compared with IGS downloaded from GenBank. Accession nos. DQ000277–DQ000287 were determined in this study.

Cultivable isolates of *B. duttonii* were identical over the 587-bp portion of the IGS sequenced and, consequently, the Ly strain was selected to represent these isolates. This isolate has been used by others as representative for *B*. *duttonii* (*2**,**20*). When applied to *B*. *recurrentis*, IGS fragment sequencing was able to group these isolates into 2 types that differed by 2 nucleotides (nt). Of the 18 isolates A1–A10, A17, and A18 comprised type I, while isolates A11–A16 gave a second type II profile. When the 2 *B*. *recurrentis* types were compared with *B*. *duttonii* types I–IV, differences appeared negligible (Table 1, Figure 1). The IGS fragment sequences of *B*. *duttonii* type II and *B*. *recurrentis* type I were identical.

**Table 1:** Sequence heterogeneity among the 568- to 587-bp intragenic spacer sequence of the Borrelia duttonii/B. recurrentis group*

	Position															
	108	129	187	215	304	325/326	379	402	438	441	456	467	474	491	525	532
Strain																
*B*. *recurrentis* type I	A	T	T	C	T	GT	T	A	T	G	T	T	A	G	G	C
*B*. *recurrentis* type II	A	T	C	T	T	GT	T	A	T	G	T	T	A	G	G	C
*B*. *duttonii* type I	G	T	C	C	C	GT	T	A	C	T	T	T	A	G	A	G
*B*. *duttonii* type II	A	T	T	C	T	GT	T	A	T	G	T	T	A	G	G	C
*B*. *duttonii* type III	A	C	T	C	T	GT	T	A	T	G	T	T	A	G	G	C
*B*. *duttonii* type IV	G	T	C	C	T	AC	G	–	T	T	C	C	G	A	G	G
Consensus sequence	A	T	T/C	C	T	GT	T	A	T	G	T	T	A	G	G	G/C

*Only exemplar sequences are included. GenBank accession nos. for *B*. *recurrentis* are DQ000277–8, while those for *B*. *duttonii* types I-IV, they are DQ000279–DQ000282.

**Figure 2:**
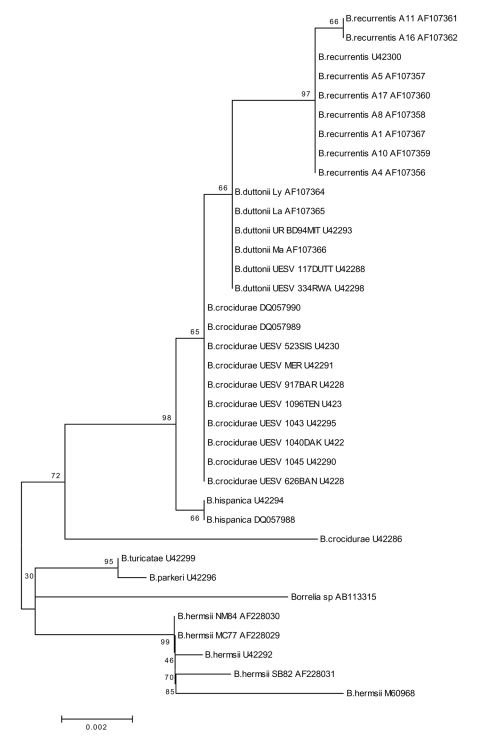
Neighbor-joining phylogenetic tree (bootstrap value 250) showing clustering of the rrs gene between Borrelia duttonii/B. recurrentis and B. crocidurae.

A comparison of *rrs* gene sequences confirmed the difference between the 2 groups of *B*. *recurrentis*, in this case, with only a single nucleotide difference (Table 2, Figure 2). Whereas the IGS fragment analysis produced different profiles within species, analysis of the *rrs* gene sequences, with the exception of the *B*. *recurrentis* types, produced single clusters for each relapsing fever species (Table 2, Figure 2). Differences between species were small, with a 4-nt difference between *B*. *recurrentis* and *B*. *duttonii*; a 6-nt difference between *B*. *recurrentis* and *B. crocidurae*; and only 2 nt differentiating *B*. *duttonii* and *B*. *crocidurae* (Table 2).

**Table 2:** Sequence heterogeneity within and between Borellia spp. using the rrs gene

Type or species	Position						
	57	206	373	488	917	1013	1429
*B*. *recurrentis* type I	A	T	C	T	T	G	T
*B*. *recurrentis* type II	A	T	C	T	T	A	T
*B*. *duttonii*	A	C	C	C	C	G	C
*B*. *crocidurae*	G	C	–	C	C	G	C

### Lice

Clothing lice collected from 21 febrile, spirochetemic louseborne relapsing fever patients from Ethiopia produced amplicons from 20 samples. Of these, 18 were identified as the *B*. *recurrentis* type I, and 2 represented type II. Thus, sequence types detected among clinical isolates mirrored those found in the lice.

### Ticks

Tick infestation rates in traditional Tembe huts approached that previously described (*1*), with ticks found in an average of 61% of the 150 huts tested (range 49%–69%). Ten of 14 Makulu village huts contained ticks positive by partial IGS PCR (71.4%), and all were identical to *B*. *duttonii* Ly strain. Ten of 11 Makang'wa village huts (91%) were similarly positive for IGS DNA. Greater heterogeneity was observed with 3 IGS types detected. The first, type I, was identical to the Ly strain and was found in 8 of the tick extracts (80%). Ticks from hut MA/15 gave a different sequence, diverging by 7 nt from the Ly strain (type II). Notably, the IGS fragment produced from these ticks was identical to that seen in *B*. *recurrentis* type I. A further profile, type III, was identified in ticks found in another hut, MA/18, which also differed in 8 nt from the Ly strain and in 1 nt from type II. Type II and III sequences were the same size as those in the Ly strain (587 bp).

Nine of 12 Iringa Mvumi village tick extracts (75%) yielded borrelial IGS fragment amplicons; 1 (IM/36) produced 2 different-sized bands (sequence types I and an IGS sequence showing greatest homology with *B*. *crocidurae*). Sequence analysis showed 4 IGS types. *B*. *duttonii* type I (Ly strain) was again predominant, identified in 7 (70%) of the 10 sequences, with the smaller (568 bp) detected as a mixed infection in 1 tick extract (IM/36, 10%). The sequence for this smaller band fell outside the *B*. *duttonii*/*B*. *recurrentis* cluster and showed greatest homology with an isolate of *B*. *crocidurae*. However, this band was surprisingly distant from the IGS *B*. *crocidurae* sequence held in GenBank AF884004. Because of its highly divergent nature, this latter sequence was excluded from further analysis. Two additional single tick extracts (each representing 10%), yielded larger amplicons of 757 bp and 759 bp, respectively (IM/16 and IM/19). Substantial differences between these sequences and those of *B*. *duttonii* were evident, and these sequences were assigned *Borrelia* spp. types 1 and 2, respectively (Table 3). Ticks from 9 (75%) of 12 huts from Ikombolinga amplified, yielding 2 distinct IGS fragment types. Ly strain type I was found in most (8 [89%]/9), and a larger amplicon of 762 bp was sequenced from IK/23, which shared little sequence homology with the Ly strain. As a result of this divergence with known *B*. *duttonii*, this amplicon was assigned *Borrelia* spp. type 3.

**Table 3:** Intergenic spacer sequence diversity among 757–762 bp Borrelia spp., DQ000284–DQ000286

Strain	Location										
	133	224	255	290	336	337	347	402–5	424	512	520
Type 1 (IM/16)	C	G	G	A	–	–	T	–	C	A	C
Type 2 (IM/19)	T	A	A	G	T	A	T	–	C	G	T
Type 3 (IK/23)	C	A	G	G	T	G	–	TAGA	T	G	T
Consensus sequence	C	A	G	G	T	X	T	–	C	G	T

### Clinical Tickborne Relapsing Fever Patients

DNA was extracted from 6 partially purified blood cultures from tickborne relapsing fever patients and amplified by using IGS fragment primers. Of these, 5 (83%) were identical to those of the Ly strain (type I), and 1 was 568 bp, identical to strains found in ticks (IM/36) and showing greatest homology with *B*. *crocidurae*. An additional 6 blood samples from spirochetemic tickborne relapsing fever patients yielded amplicons, 5 of which were identical to the Ly strain, while the remainder, *B*. *duttonii* type IV (patient WM) showed 10–12 nt differences and 1 nt deletion when compared with other sequence types (Table 1).

In summary, *B*. *duttonii* type I was predominant, found in isolates from patient isolates and blood from patients with clinical cases of tickborne relapsing fever and *O*. *moubata* ticks. In fact, this was the only type represented among cultivable isolates. Three variants, types II, III, and IV, were occasionally found in both clinical patients (type IV) and ticks (types II, III). A further, smaller, amplicon showed greatest homology with *B*. *crocidurae* and fell outside the cluster of *B*. *recurrentis* and *B*. *duttonii* sequences and was found in both a tick and a patient. Although these variants are an apparent minority, they have been found in conjunction with type I in ticks (*B*. *crocidurae*–like) and as the only *Borrelia* type found in patients with clinical cases (1 patient with *B*. *duttonii* type IV and 1 with a *B*. *crocidurae*–like spirochete). Whether these strains can be cultivated remains unresolved.

Novel partial IGS sequence types were found in ticks from huts IM/16 and IM/19, which clustered together with those found in IK/23 and were assigned *Borrelia* spp. types 1, 2, and 3, respectively. Each of these *Borrelia* types showed slight variability in their IGS fragment sequence (Table 3); however, they formed a distinct cluster away from other borrelial species. Insufficient material remained to undertake further PCR assays with other targets such as the *flaB* or *rrs* genes. Greatest homology was with Nearctic species *B*. *hermsii* and *B*. *turicatae*. All differ substantially from the Ly *B*. *duttonii* strain, except over the primer regions, and consequently cannot be considered as belonging to this species.

### Accession Numbers

*B*. *recurrentis* types I and II were assigned GenBank accession numbers DQ000277 and DQ000278, respectively. *B*. *duttonii* groups I–IV were assigned DQ000279-DQ000282, respectively. The *B*. *crocidurae*–like sequence was given DQ000283. *Borrelia* spp. IGS sequences types 1–3 were assigned DQ000284-DQ000286, respectively, while the *B*. *crocidurae* IGS sequence determined in this study was given DQ000287.

## Discussion

Application of gene sequence–based approaches has provided useful information regarding population structure and possible phylogenetic associations among *Borrelia* spp (*21*). Of particular value has been sequence-based comparisons of the *rrs*-*rrlA* IGS region, which (because noncoding DNA shows greater variability than would be expected for coding sequences) is sufficiently conserved to permit phylogenetic conclusions (*22**,**23*). Our results demonstrated that the differences between *B*. *duttonii* and *B*. *recurrentis* were as great as those within species, raising the question of whether these spirochetes are indeed a different species. This difference is exemplified by the identical IGS fragment sequence shared between *B*. *recurrentis* and *B*. *duttonii* type II. Whether these data suggest a common ancestral lineage for these relapsing fever spirochetes can only be fully resolved through sequence analysis of the whole genome. An analogous situation was found when IGS typing was used to characterize other microbes such as *Fusobacterium* spp.; in that situation, good resolution of strains was provided; however, with IGS typing, 2 species appeared to be identical (*23*).

Analysis of *rrs* phylogeny, in contrast, showed distinct clusters for *B*. *duttonii*, *B*. *recurrentis*, and *B. crocidurae*; however, a small number of nucleotides differentiated these clusters. Some Old World relapsing fever spirochetes show a high degree of similarity, both in using 16S RNA gene sequences (*12*) and in flagellin (*20**,**24*). Using the 16S RNA gene, we found that only 4 bases differed over the *rr*s gene between *B*. *duttonii* and *B*. *recurrentis*; this finding was also reported previously (*12*). Ras et al. concluded that this small difference did not support these being single species but instead postulated that *B*. *recurrentis* could be a clone derived from the *B*. *crocidurae–B*. *hispanica–B*. *duttonii* cluster (*12*). Although our results would support the concept that *B*. *recurrentis*, *B*. *duttonii*, and *B*. *crocidurae* are clonal variants, IGS fragment typing suggested a substantially greater distance between the *B*. *duttonii*/*B. recurrentis* complex and *B*. *crocidurae*. More *B*. *crocidurae* isolates were not available to further verify phylogenetic inference by using partial IGS sequencing. A further bias within this comparison is that the *rrs* phylogeny presented here used cultivable isolates. Since only *B. duttonii* type I was represented among these isolates, the *rrs* gene phylogeny may not truly represent the diversity found among these spirochetes.

Despite these apparently conflicting datasets, resolution of the IGS fragment typing was clearly greater, enabling a more detailed analysis of clinical or host associations. Failure to discriminate between the *B*. *duttonii*/*B*. *recurrentis* complex, however, would require the use of alternative targets, if they are indeed different species. This apparent paradox may reflect that these are clones derived from a common ancestral origin (*12*). This hypothesis is strengthened by the finding of a partial IGS sequence (*B*. *duttonii* type II) in *O*. *moubata* complex ticks from a disease-endemic area for tickborne relapsing fever, which showed total homology with the predominant sequence type identified among *B*. *recurrentis*. Further investigation of ticks from this area should be conducted.

To overcome problems associated with use of single gene targets for addressing population structure among microorganisms, multilocus sequence typing (MLST), which is less likely to show linkage disequilibrium than many surface markers, is commonly used to investigate more conserved housekeeping genes. However, a few housekeeping genes possessed by *Borrelia* spp. are located on plasmids, which raises a question about the validity of this approach (*25*). Others have used surface exposed proteins as targets for MLST (*14*). More recently, an MLST study focused on 18 different, highly polymorphic loci to show the role of recombination in creation of diversity among Lyme disease–associated spirochetes and their correlation with outer surface protein C alleles (*25*). Despite extensive application of these methods to Lyme disease–associated *Borrelia* spp., the relapsing fever spirochetes have been largely neglected. However, recently a MLST-based approach, in which *rrs*, *flaB*, *gyrB*, and *glpQ* chromosomal genes were used to examine the phylogenetic relationship among *B*. *parkeri* and *B*. *turicatae*, confirmed their species status and demonstrated that the Florida canine *Borrelia* isolate clustered among *B*. *turicatae* (*26*).

Investigations have been limited by the lack of available strains for assessing and identifying suitable markers. Cultivating these fastidious microbes is challenging, with some, until recently, considered noncultivable (*16**–**18*). Furthermore, the complexity of coexistent mechanisms for antigenic variation with associated potential for major genomic reorganization presents a problem for many microbiologic typing approaches. Isolates that are homogeneous by gene sequence–based approaches may present vastly different pulsed-field electrophoretic profiles (*16**,**17**,**26*), possibly through extensive genetic duplication associated with antigenic variation of major outer membrane proteins, whose genes are carried on large linear plasmids (*7**,**11*).

Although using the more variable IGS region for molecular typing of relapsing fever spirochetes has been reported as valuable (*15*), only a limited selection of relapsing fever species, *B*. *miyamotoi* and *B. lonestari*, have been assessed. When we used this approach to characterize clinical isolates from patients with tickborne relapsing fever and louseborne relapsing fever, blood samples from patients, and ticks and lice collected in East Africa, we demonstrated that this method could successfully be applied to all sample types. We also demonstrated 3 distinct groups: a) 1 comprising cultivable strains of *B*. *duttonii/B*. *recurrentis* and amplicons from tick, lice, and human blood samples; b) 1 from both a patient and a tick showing greatest homology with *B*. *crocidurae*; and c) a further group containing unique amplicons from ticks collected in Tanzania. This last group was distinct in its IGS sequence and is likely compatible with the novel *Borrelia* species from this region (*3**,**4**,**20*). Three sequence types were identified that clustered within this novel IGS sequence clade, separate from the *B*. *duttonii*-*B*. *recurrentis* complex. This clade fell among those containing *B*. *hermsii* and *B*. *turicatae* (Figure 1), thus supporting earlier reports of a new *Borrelia* spp. characterized by using flagellin and 16S RNA sequences (*Borrelia* species AB113315 [3], Figure 2), and reported to show greater resemblance to Nearctic isolates such as *B*. *hermsii* and *B*. *turicatae*, rather than Old World tickborne relapsing fever and louseborne relapsing fever isolates (*3**,**4**,**20*). Insufficient material remained to allow repeat analysis for either *flaB* or *rrs* genes to confirm that *Borrelia* species types 1–3 were indeed the same spirochete described above. These *Borrelia* spp. were not detected in any relapsing fever patient samples; consequently, their clinical significance has yet to be established.

Of further interest was the identification of a *B*. *crocidurae*–like sequence in a patient and *O*. *moubata* tick. *B*. *crocidurae* is not believed to be present in East Africa but is endemic to the West African coast. Although the patient may have traveled from the west coast, finding this sequence type in a tick that was co-infected with the local endemic *B*. *duttonii* suggests that this spirochete may coexist in this area and may have extended vector compatibility. These findings were facilitated by methods able to give substantial resolution between strains, such as partial IGS typing, and would not have been possible by using clinical criteria or customary diagnostic approaches.

Traditionally, relapsing fever nomenclature was assigned according to the spirochete's arthropod vector and the host species susceptibility. Clinical differences have also been documented: the louseborne form of the disease has been considered the most severe (*27**,**28*), although more frequent relapses are associated with the tickborne form. Such differences have lately been questioned because more recent studies of louseborne relapsing fever suggest less severe outcomes (*29*). *B*. *duttonii* has been correlated with higher perinatal death rates (*30**–**32*), while *B*. *recurrentis* is associated with a higher incidence of epistaxis (*28*). These differences suggest that the diseases are associated with different clinical etiologic agents; however, these differences may actually be associated with differences in transmission or even in host response. Alternatively, different clinical symptoms may be serotype associated. Indeed, serotype switching has been associated with different clinical symptoms in rodent models infected with the American relapsing fever spirochete, *B*. *turicatae* (*33*). The lack of animal models for East African isolates precludes similar studies among these spirochetes. However, different isolates of the same species (*B*. *recurrentis*), which express different variable membrane proteins, have been shown to induce tumor necrosis factor release to different degrees (*34*).

Unlike many relapsing fever spirochetes, *B*. *duttonii* and *B*. *recurrentis* infections are believed to be diseases of humans with as yet unidentified animal reservoirs. This restricted host range may impose constraints on these species compared with their less host-specific relatives. Indeed, this could account for greater diversity among other tickborne *Borrelia* spp. than among *B*. *duttonii* and the louseborne *B*. *recurrentis*.

In summary, 38 *B*. *recurrentis* sequences and 47 *B*. *duttonii* IGS fragments were studied, showing 2 sequence types differing by 2 nt in the former and 4 types differing in from 1 to 12 nt (and a further deletion in 1 type) in the latter. These observations suggest more recent evolution of *B*. *recurrentis*, if one assumes that both spirochetes acquired changes in their IGS region at the same rate. IGS fragment sequencing proved to be a valuable typing tool for these spirochetes, allowing discrimination between relapsing fever and other borrelial species carried by ticks and lice prevalent in the same geographic region. However, this method was unable to separate *B*. *duttonii* and *B*. *recurrentis*. This typing approach appeared sufficiently robust to work on multiple sample types, including isolated strains, patient blood samples, and ticks and lice, thus negating the requirement for cultivation.
